# Hypoplasia of Depressor Angularis Oris in a Male Neonate Associated with Cephalohematoma

**DOI:** 10.7759/cureus.7213

**Published:** 2020-03-08

**Authors:** Dominic Parfianowicz, Uchendu Uchendu

**Affiliations:** 1 Internal Medicine, Philadelphia College of Osteopathic Medicine, Philadelphia, USA; 2 Pediatrics, Reading Hospital-Tower Health, West Reading, USA

**Keywords:** asymmetric crying facies, congenital hypoplasia, depressor angularis oris

## Abstract

Congenital hypoplasia of depressor angularis oris muscle (CHDAOM) is an uncommon cause of asymmetric crying facies in neonates. Although its etiology is mostly unknown, it has been increasingly recognized as a marker for the presence of other less easily identifiable congenital abnormalities associated with genetic syndromes such as DiGeorge and Cayler syndrome. We report a unique case of a male neonate that highlights the necessity of judicious and accurate clinical documentation with the presence of CHDAOM to avoid unnecessary forms of subsequent work-up.

## Introduction

Asymmetric crying facies (ACF) is characterized by unilateral facial weakness of the lower lip which becomes prominent especially when the infant is crying. Having been reported as often as one in 160 live births, ACF is not particularly rare [1]. It can occur due to nerve compression, as seen in the setting of birth trauma, or less often due to congenital defective muscle development [2,3]. There are several muscles involved in depressing the lower lip, all of which are innervated by the marginal mandibular branch of the facial nerve. Of these lip depressors, unilateral congenital hypoplasia of depressor angularis oris muscle (CHDAOM) has been most commonly implicated in the setting of ACF [2].

## Case presentation

**Figure 1:**
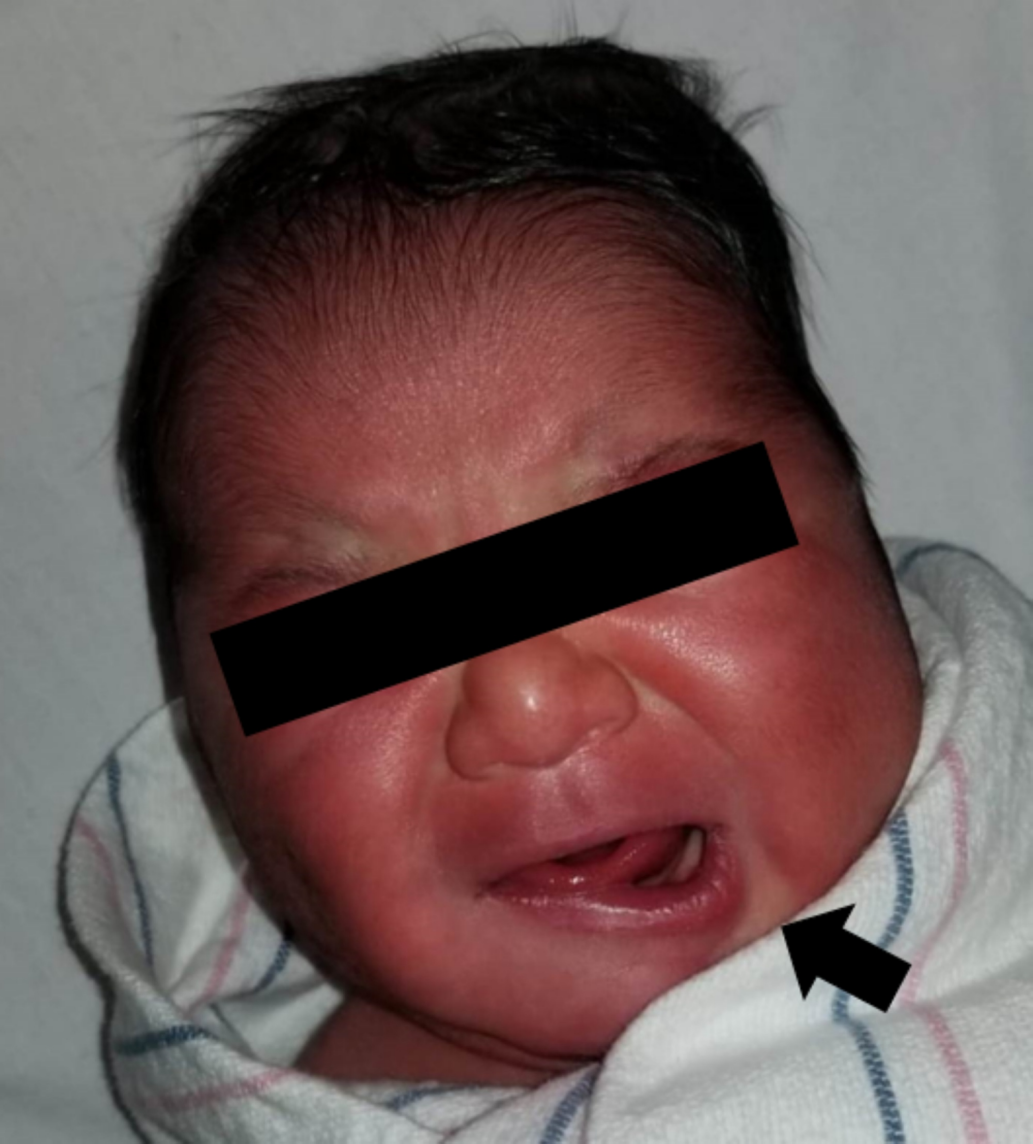
Facial Asymmetry with Crying Photograph taken during evaluation at 28 hours since birth showing facial asymmetry in the neonate while crying. Drooping of the left lower lip with apparent shift toward the left side is seen most likely as a result of a hypoplastic depressor angularis oris muscle on the right side.

**Figure 2:**
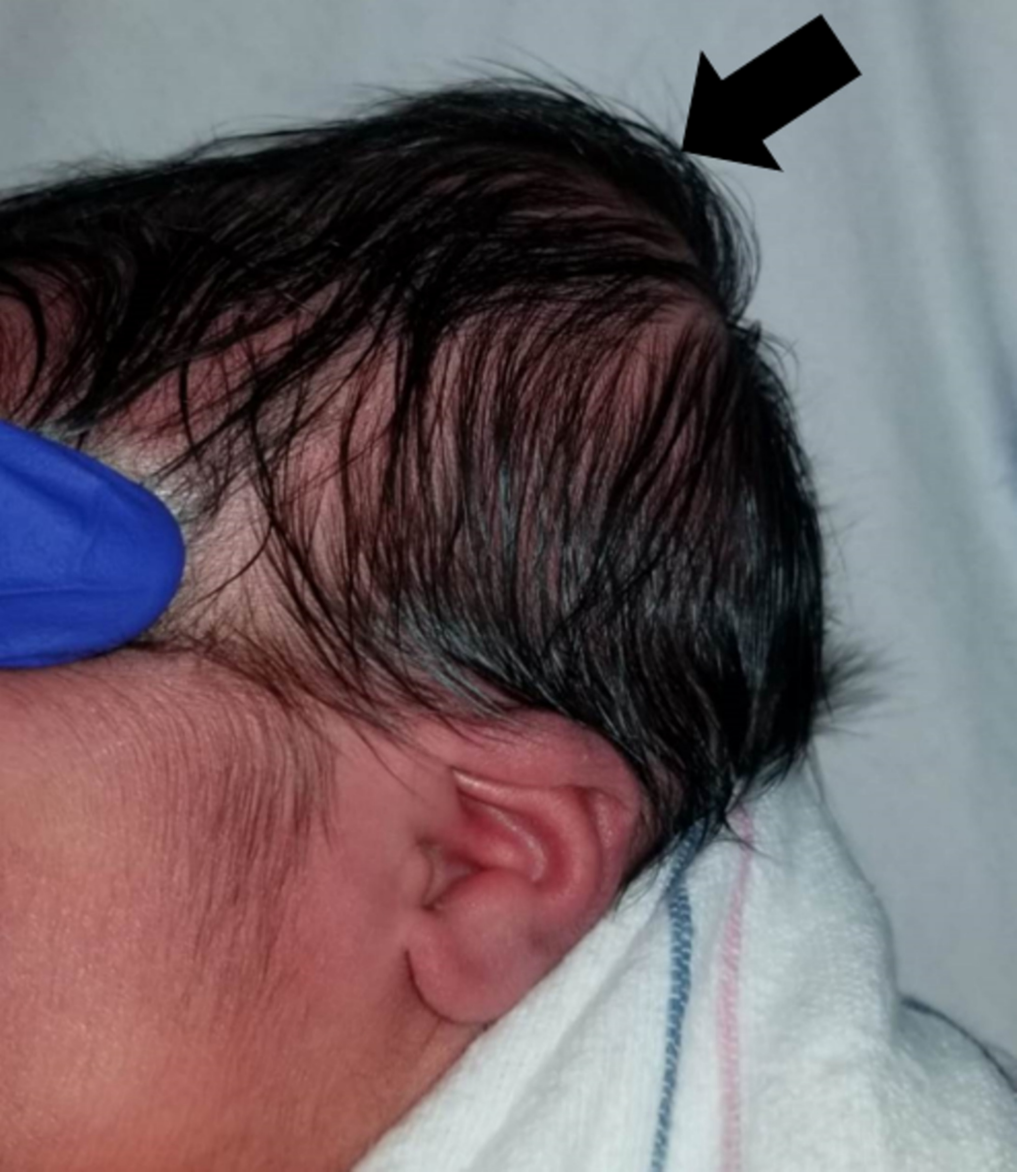
Left Posterior Cranial Swelling Photograph taken during evaluation at 28 hours since birth showing a well-demarcated swelling in the left posterior parietal/occipital region.

**Figure 3:**
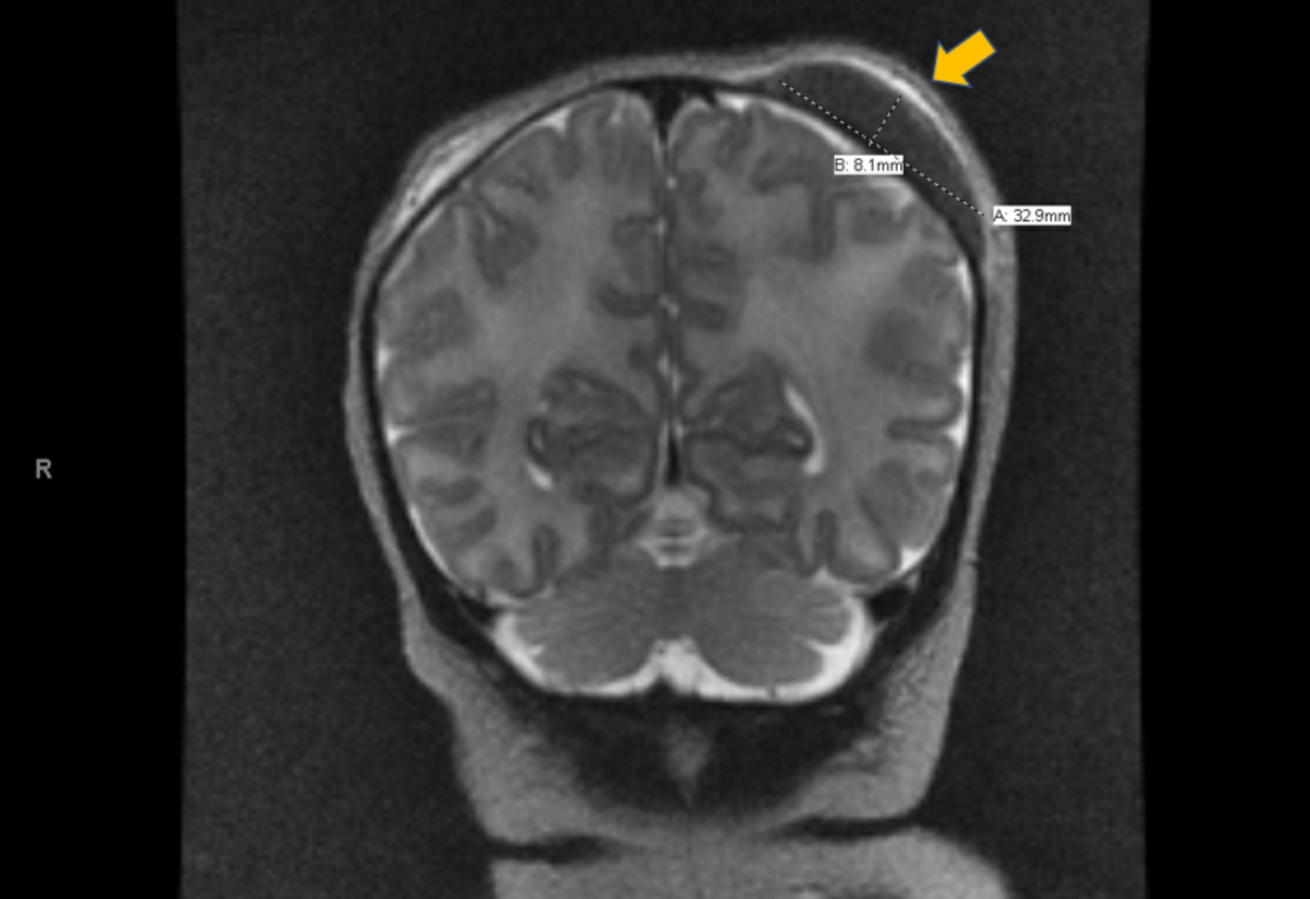
Non-contrast MRI: Coronal View A non-contrast MRI in coronal view showing the presence of a cephalohematoma with no intracranial lesion identified.

**Figure 4:**
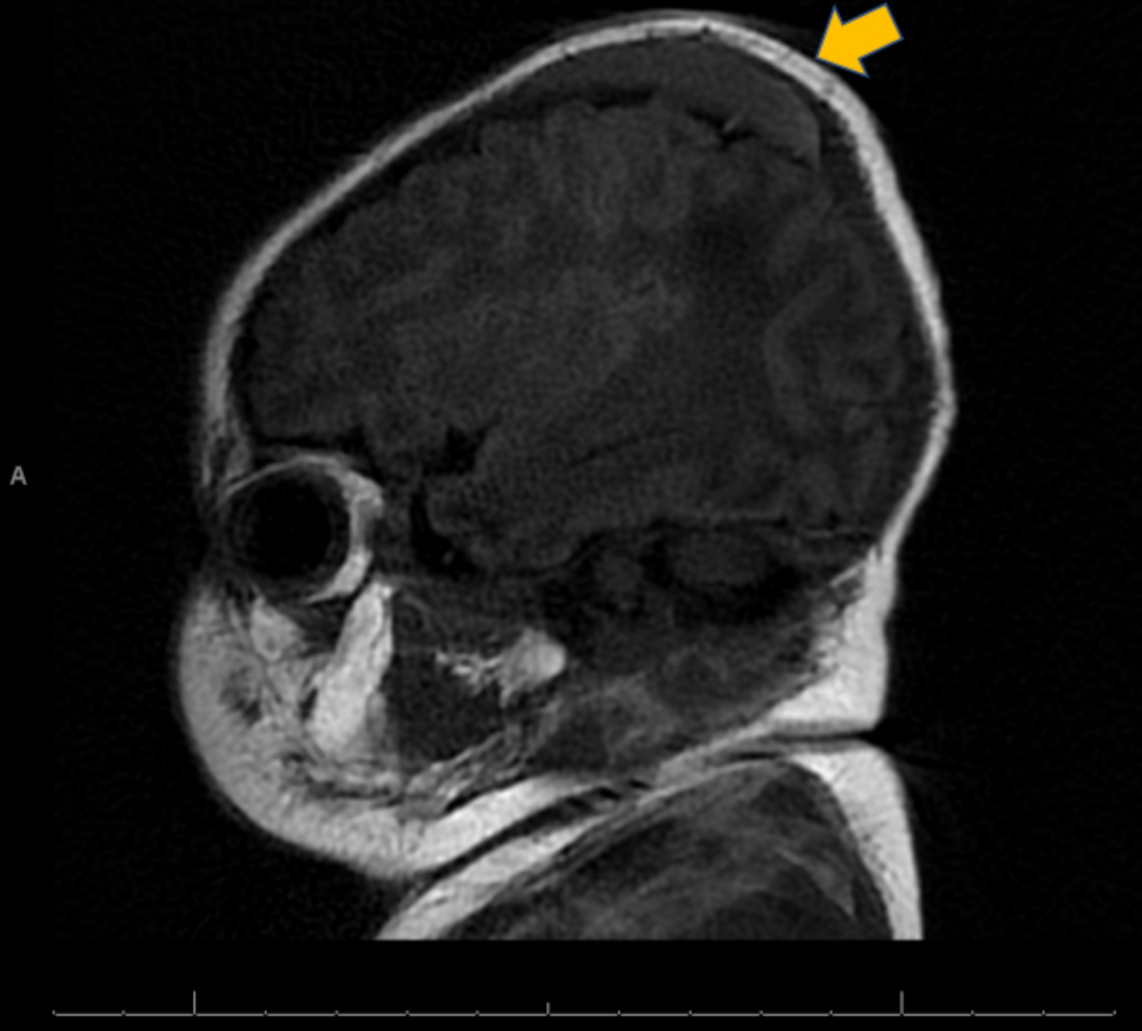
Non-contrast MRI: Left Sagittal View A non-contrast MRI in a left sagittal view showing the presence of a cephalohematoma with no intracranial lesion identified.

A male neonate was born via low transverse Cesarean section to a 27-year old gravida 2, para 2 mother at 39 weeks and zero-day gestation. Apgar scores were 8 and 9. Past medical history and family history were noncontributory. The prenatal and perinatal periods were uneventful, and the nursing staff expressed that no instrumentation was used for delivery that may have resulted in a known birth trauma. Deviation of the lower lip to the left side while crying, suggesting right-sided muscle weakness, was noted at the time of delivery (Video 1, Figure 1). This, however, did not interfere with the boy’s ability to latch or breastfeed. Initial physical examination was otherwise in accordance with delivery of a healthy male neonate with no focal neurological deficits. Follow-up examination at 28 hours was unchanged except for a new well-demarcated swelling of the left posterior parietal region (Figure 2). Further clarification was sought regarding the use of instrumentation during delivery as the swelling strongly suggested the presence of cephalohematoma. The appearance of a potential left-sided cephalohematoma along with right-sided lower lip weakness and no obvious birth trauma raised concern for other potential congenital malformations, including the remote possibility of an intracranial lesion. Non-contrast magnetic resonance imaging (MRI) of the head confirmed cephalohematoma with no intracranial abnormalities (Figures 3, 4). The patient was later discharged at 53 hours of life after passing both hearing and critical congenital heart disease screenings.

 

**Video 1:** Facial Asymmetry with Crying Video taken during evaluation at 28 hours since birth of a male neonate with asymmetric depression of the lower left lip and shift to the left side while crying with return to a normal symmetric face when calm. This is likely due to congenital hypoplasia of depressor angularis oris muscle (CHDAOM).

The patient was diagnosed with right-sided ACF due to CHDAOM along with an idiopathic cephalohematoma. At the time of discharge, the parents elected to receive care at an affiliated primary care office in the locality. In communicating with the providers at the affiliated health facility, the report was that the baby had remained clinically stable. The facial asymmetry showed progressive but slow improvement, and as previously noted it did not seem to affect the patient’s ability to feed normally. He had regained birth weight by the two-week well child visit.

## Discussion

ACF is a broad term describing a unilateral lower lip asymmetry that becomes evident in a crying neonate but is symmetric while at rest. Nerve compression and abnormal muscle development have been suggested as possible causes [3]. Compression of the peripheral roots of the facial nerve commonly results from birth trauma or abnormal intrauterine fetal lie [2]. Furthermore, impingement of the marginal mandibular branch of the facial nerve which innervates several lip depressors may produce an ACF-like presentation. Termed congenital facial paralysis, the resulting facial asymmetry is an isolated finding and an overwhelming majority of these cases tend to have complete resolution [1,2]. CHDAOM is another cause of ACF but is particularly rare. Although its pathogenesis is not well described, viral infection during pregnancy and familial heredity have been speculated to have some causative influence [2,3]. The DAOM attaches from the mandible to the corner of the mouth, functioning to lower the corresponding side of the mouth. In the setting of unilateral CHDAOM, the lower lip appears symmetrically horizontal at rest while appearing to be elevated on the hypoplastic side during crying or frowning as compared to the normal side. This is due to an inability to depress the side ipsilateral to the hypoplastic DAOM.

CHDAOM can present as an isolated finding but also can occur with other congenital malformations in up to 45%-70% of cases [3-5]. These malformations are not limited to any particular body system, but most commonly involve the head and neck regions and cardiovascular system [4,5]. Our patient had swelling of the left posterior parietal area of the head contralateral to the affected side of the mouth (right). The exact onset of this swelling was not known by the nursing staff but was highlighted during rounding by the attending provider on the second day of life. This raised suspicion for a possible contralateral intracranial lesion as a cause of the observed ACF, given the unclear history of instrumentation at delivery. Evaluation with MRI confirmed the presence of an idiopathic cephalohematoma (Figure 3). When CHDAOM is seen among a collection of other exam findings suggesting congenital disease, it is most notably associated with syndromes such as DiGeorge syndrome, VACTERL (Vertebral anomalies, Anal atresia, Cardiac defects, Tracheo-Esophageal fistula, Renal anomalies, Limb defects) association, and Cayler cardiofacial syndrome among others [4-6]. CHDAOM itself is a clinical diagnosis and is a benign condition when occurring in isolation, yet it has been suggested that the diagnosis of CHDAOM may be an indicator of concomitant congenital abnormalities [1,4,6]. Therefore, thorough physical examination and comprehensive newborn screening must be performed to either rule out any of the aforementioned syndromes or to begin prompt treatment and correction of the more serious coexisting abnormalities.

## Conclusions

In this index case, we considered the need for imaging to rule out a possible associated congenital anomaly given the presence of the head swelling, which had been suspected to be a cephalohematoma and did not really require an imaging study, such as an MRI to confirm. Our case underlines the need for accurate clinical documentation in helping to delineate an otherwise relatively rare clinical condition without the involvement of unnecessary expensive imaging or other forms of work-up.
